# Development and Validation of Positive Smoker Identity Questionnaire (PSmoQi): A New Instrument for Smoking Cessation Correlates

**DOI:** 10.3390/ijerph16030351

**Published:** 2019-01-26

**Authors:** Mohd Hanief Ahmad, Mohd Ismail Ibrahim, Azriani Ab Rahman, Kamarul Imran Musa, Faridah Mohd Zin, Rehanah Mohd Zain, Ruhaya Hasan, Noraryana Hassan, Imran Ahmad, Nur Suhaila Idris

**Affiliations:** 1 Department of Community Medicine, School of Medical Sciences, Universiti Sains Malaysia, Kubang Kerian, 16150 Kelantan, Malaysia; drhanief@moh.gov.my (M.H.A.); azriani@usm.my (A.A.R.); drkamarul@usm.my (K.I.M.); 2 Department of Family Medicine, School of Medical Sciences, Universiti Sains Malaysia, Kubang Kerian, 16150 Kelantan, Malaysia; faridahz@usm.my (F.M.Z.); profimran@usm.my (I.A.); nursuhaila@usm.my (N.S.I.); 3 Hospital Universiti Sains Malaysia, Kubang Kerian, 16150 Kelantan, Malaysia; 4 School of Health Sciences, Universiti Sains Malaysia, Kubang Kerian, 16150 Kelantan, Malaysia; rehanah@usm.my; 5 School of Dental Sciences, Universiti Sains Malaysia, Kubang Kerian, 16150 Kelantan, Malaysia; ruhaya@usm.my; 6 FCTC and Tobacco Control Unit, Disease Control Division (NCD), Ministry of Health Malaysia, 62590 Putrajaya, Malaysia; noraryana@moh.gov.my

**Keywords:** smoking cessation, positive smoker identity instrument, questionnaire development, validation, reliability

## Abstract

**Background:** The positive smoker identity construct, which was based on West’s PRIME Theory, affected the smoking prevalence, quit attempts and cessation success. A validated questionnaire which could measure this rich and complex construct may facilitate prediction models of successful cessation. We aimed to develop and validate a questionnaire that assesses positive smoker identity based on West’s PRIME Theory. **Method:** The initial item pool was developed based on a theoretical framework, empirical literature, existing scales and expert review. The questionnaire was conveniently distributed to 100 smokers. Exploratory factor analysis was utilized to explore domains in the questionnaire. Construct and criterion validity, internal consistency and reliability of the domains were analyzed. **Results:** The final positive smoker identity questionnaire (PSmoQi) has 26 items under four internally-consistent and reliable domains: Contributory factors, contextual and temporal patterning, identity related to smoking, and behaviour in relation to smoking. The full scale demonstrated good internal consistency (∝ = 0.78), acceptable convergent and divergent validity, and good concurrent validity with the smoker self-concept scale. **Conclusion:** The current study provides fundamental evidence for the PSmoQi as a valid instrument in research related to smoking cessation and interventional strategy. The PSmoQi contained validated domains which could measure almost a full spectrum of smoking cessation components.

## 1. Introduction

### 1.1. Theories in Smoking Cessation

Smokers’ readiness and motivation to quit have been studied and been shown to be important factors in cessation success [1,2]. Smoking cessation programs have incorporated many theories into practices, for example the transtheoretical stage of change model [3,4], social cognitive theory [5,6], protection motivation theory [7,8], health belief model [9], theory of planned behaviour [10,11], and social-ecological model [12]. However, there were mixed results in terms of the efficacy of the cessation intervention programs which were anchored in these theories [13,14,15,16], not to mention the critiques towards and limitations of these theories [17,18,19,20].

### 1.2. PRIME Theory

**Figure 1:**
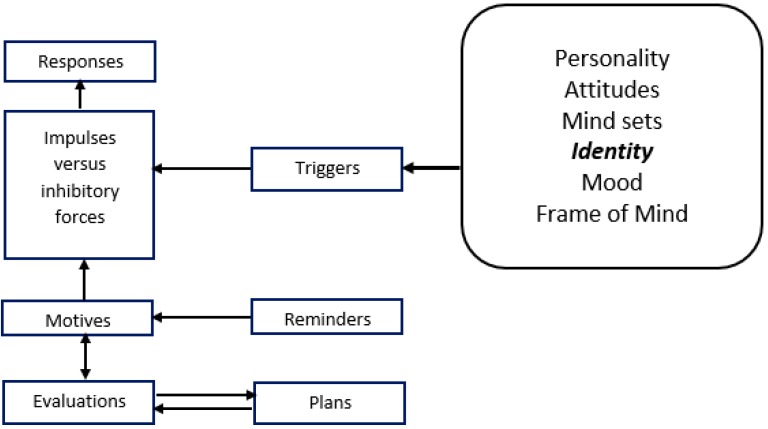
Theoretical framework of PRIME Theory.

West’s PRIME Theory is one of the newest theories which explain behaviours, especially addictive ones, using a dynamic model. This theory (refer to Figure 1) explains the complexity of why people continue or stop smoking using five levels of motivational system including responses, impulses, motives evaluations and plans [21]. West defined identity as “thoughts and images of ourselves and how we feel about these”. Thoughts are classified into “labels” (the categories to which we consider that we belong, e.g., smoker), “attributes” (the features we ascribe to ourselves, e.g., rebellious), and our “personal rules” (the things that we do and do not do, e.g., not smoke indoors). Identity is part of the mental representations of ourselves and the feelings attached to these. Identity is a potentially important source of motives, is the ultimate source of self-regulation, and is a major source of stability of behaviour.

### 1.3. Smoker Identity Construct

Little research has been published on smoker identity. It has been shown that both adults [22] and young smokers [23] reported shifting between different smoker identities (e.g., from smoker to non-smoker) during the process of cessation. There was also some evidence that smokers made efforts to distance themselves from their unwanted smoker identity [24,25]. But often this identity transition was not sufficient to achieve long-term abstinence, and they can carry on smoking secretly [26] or occasionally [24,25]. Young smokers with a strong non-smoker identity were more likely to remain abstinent when compared with heavy smokers with an established smoker identity, even though they also reported negative feelings about smoking and being a smoker [23].

Quantitative studies suggested potential discrepancies between smoker identity and behaviour, that is, despite smoking cigarettes people denied being a smoker [27,28,29,30]. Those denying their smoker identity tended to be younger, male [27], to smoke occasionally [29], and to not have made an attempt to quit in the past year [27]. There is some evidence that having developed a smoker identity is associated with smoking escalation in adolescents [31] and resistance to anti-tobacco messages [32,33]. Smokers with a smoker identity were found, in two studies of specific groups of smokers, to be less likely to intend to [32] and make a quit attempt [34]. Moreover, smoker self-concept and abstainer self-concept at baseline were reported to be important factors in predicting the success of smoking cessation treatments among adults [35]. This smoker self-concept was derived from smoker self-schema which differentiated them from non-smokers, would drive them to heighten sensitivity to smoking signal, impulses to smoke, probability of smoking and likelihood of quitting. The abstainer self-concept was deduced from abstainer possible-self in non-smokers that the smoker aimed to achieve and would encourage non-smoking behaviour and quitting. Having a positive smoker identity, as measured by agreement with the statement “I like being a smoker”, was associated with being older, male, reporting stronger nicotine dependence, lower motivation to stop smoking, and not having made a quit attempt in the past year [36].

### 1.4. Research Gap

Most studies on smoker identity were qualitative and those quantitative studies used a single “yes or no” question as an indication of whether a smoker had a positive smoker identity or not. Thus, this measure will not capture the complexity and richness of this construct. To the best of our knowledge, there is no established and validated positive smoker identity measure available in the Malay language and the Malaysian population at the moment. This is very important because the culture and belief will determine the behavior of the smokers. The newly developed questionnaire will serve as a tool for similar patterns of community, especially in the South-East Asia region. Table 1 shows studies which explored the smoker identity construct and the question(s) used to represent the construct.

Shadel and Mermelstein [35] validated a five-item smoker self-concept scale, which measured the self-concept construct in social cognitive conception with an alpha-coefficient of 0.74. The self-concept of social cognitive conception may be similar with the positive smoker identity construct of PRIME theory. However, the sample, population, place, time, scenario and situational circumstances of the study were totally heterogeneous in the current study.

The current study questionnaire could facilitate the matching of smokers with strategies and interventions that are more likely to help them quit, and to make the most of health care resources. A study [39] showed the average costs per quitter, per patient and per quit attempt were MYR 953.28 (USD 308), MYR 55.71 (USD 18) and MYR 34.74 (USD 11), respectively. This cost could be reduced substantially if we could identify smokers who were more likely to stop smoking and prioritized smoking cessation programs accordingly in order to achieve better effectiveness and efficiency.

### 1.5. Research Goals

The objective of this study was to obtain additional information so that the researchers can further improve the survey questionnaire. Improvements could possibly be in the factor structure of the items, dimensionality/grouping of items, and reduction of number of items. Our objective was also to test the internal consistency for the first time. We also wanted to determine the relationship between the positive smoker identity questionnaire (PSmoQi) with an existing validated and translated measure for smoking dependence (Fagerström test for nicotine dependence—FTND), for self-efficacy to avoid smoking in various situations (cessation self-efficacy questionnaire—CSEQ), and the smoker self-concept scale (SSCS). The relationship between PSmoQi with FTND and CSEQ would determine the concurrent validity of PSmoQi in smoking cessation, whilst the relationship between the PSmoQi and the SSCS would determine the concurrent validity of positive smoker identity construct with smoker self-concept construct.

### 1.6. Preliminary Studies and Questionnaire Development 

**Figure 2:**
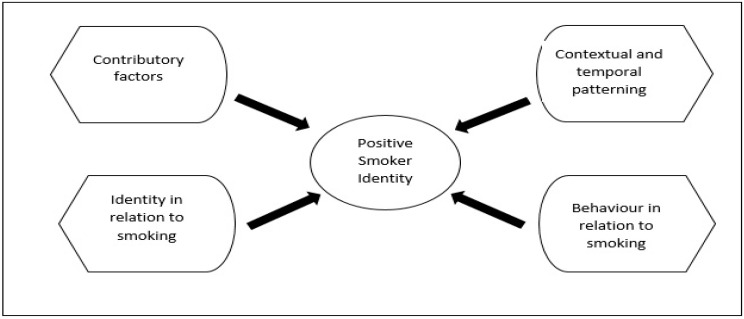
Four domains under the positive smoker identity construct. First factor: Contributory factors that lead to cigarette smoking. Second factor: Contextual and temporal patterning, which reflects the dynamic characteristic of smoking behaviour. Third factor: Identity in relation to smoking, which identifies self-categorization of a smoker. Fourth factor: Behaviour in relation to smoking.

We produced an initial collection of question items based on a comprehensive review of empirical and theoretical literature, existing scales and expert opinions. Five experts, including a tobacco questionnaire expert, a smoking cessation specialist, a health promotion and health management specialist, a family health expert and a questionnaire validation and statistic specialist, contributed to the development of the questionnaire. The initial item pool was derived from a meta-ethnography study [40]. It contained 20 items in four domains (Figure 2):

A further extensive review of other qualitative and quantitative literatures [31,32,35,36,37,38], and expert viewpoints had increased the number of items to 75. These 75 items represented the preliminary group of question items within the above four domains (Section A, B, C and D) plus a one-item domain (preparedness to stop smoking), namely Section E [38,41].

### 1.7. Face and Content Validation

During the face and content validation process, 36 of the 75 preliminary items were modified, for example, by replacing the sentences with more suitable words or by rephrasing of the sentences to improve the clarity of the sentences in the questionnaire. Four items were eliminated due to unacceptable scores in the Item Content Validity Index (I-CVI), Content Validity Ratio (CVR), Item Impact Score (IIS) and Modified Kappa. These four questions (Table 2) scored low on these four indicators due to their lack of clarity, lack of relevance to the domains and to the positive smoker identity construct, lack of importance in terms of the related domains, and also lack of completeness in relation to the wordings, sentences and syntax in the Malay language. After eliminating these four items, we arrived at the final collection of 71 questions. They corresponded to the five domains under the umbrella of the positive smoker identity construct, including a one-item domain of preparedness for smoking cessation (Section E).

## 2. Materials and Methods 

### 2.1. The Participants

The participants comprised a convenience sample of 100 adult smokers from Universiti Sains Malaysia (USM) Hospital staff and from the people who attended USM Hospital for many reasons. These people were either relatives of the patients admitted to the hospital, a well patient who was just picking up an outpatient drug prescription from the pharmacy in the hospital, a hawker in the hospital compound, or a bystander or passer-by who happened to be around the hospital compound. The sample area (located in the state of Kelantan, on the east coast of Peninsular Malaysia) and sample pool were chosen because of their demographic diversity and heterogeneous population in which male smokers from all walks of life and positions in society could be located. A smoker was defined as a person who had smoked at least 100 cigarettes or all types of nicotine product such as vape, e-cigarette, shisha or conventional cigarette in their lifetime and who had smoked at least once in the last six months. Data were collected from May 2017 to September 2017 in the presence of a researcher. All participants gave informed and written consent in this study. For the sample size calculation, we used the rule of 100 [42] for the exploratory factor analysis (EFA).

### 2.2. Research Tools

Apart from 71 items in the PSmoQi questionnaire, our study tools included a proforma containing socio-demographic characteristics (such as age, gender and job status); detailed smoking status (such as type of cigarette, number per day or per week, age of smoking initiation, and age at which smoking become daily occurrence); quit attempts data (such as number of attempt in the past year, methods used to quit and length of abstinence the last 3 attempts); self-rated health status (such as presence of co-morbidities); and awareness about anti-cigarette information in the media.

For 71 items of the PSmoQi questionnaire (except Section E), the participants’ responses were measured using a 5-point Likert scale ranging from “strongly disagree” to “strongly agree”. Section E item comprised 7 choices of response: 1) I never give a thought to stop smoking, 2) I do not want to stop smoking, 3) I gave a thought to stop smoking, but I do not want to stop, 4) I want to stop smoking but I do not know when I will stop, 5) I want to stop smoking and I am reducing the number of cigarettes I use, 6) I want to stop smoking and I am finding ways to stop, and 7) I want to stop smoking now. 

The Fagerström test for nicotine dependence (FTND) to measure nicotine dependence was also used in the study [43]. The Malay version of the Fagerström test for nicotine dependence has been validated and revealed good internal consistency with the overall Cronbach’s alpha of 0.67 [44]. FTND scores ranged from 1 to 10, of which higher scores reflected higher dependency.

The CSEQ was a 12-item questionnaire which assessed participants’ self-efficacy to avoid smoking in various situations described in each item. The translated Malay version of this questionnaire demonstrated good internal consistency with Cronbach’s alpha of 0.90 and good test–retest reliability (*r* = 0.80) over 2 weeks [45]. CSEQ scores ranged from 12 to 60. Higher scores implied higher self-efficacy. SSCS was a five-item smoker self-concept scale which measured the self-concept construct in social cognitive conception with an alpha-coefficient of 0.74 [35]. 

### 2.3. Data Analysis

Data were analyzed using SPSS software version 22. We used exploratory factor analysis (EFA) to explore possible factors in the pool of items. In order to determine factorability of the data (i.e., the appropriateness of factor analysis), the correlation matrices, the Kaiser–Meyer–Olkin Measure of sampling adequacy (KMOMSA), and the squared multiple correlation for each item were determined. A minimum acceptable score for KMOMSA test is 0.5 [46]. A Principal Components Analysis (PCA) was used as the method of factor extraction. Factors with eigenvalues greater than 1 in the factor extraction process were demonstrated. Cattell’s scree test was carried out for factor solution suggestion. The method of rotation depended on the degree of correlation between the factors. An item would be retained on a given factor if the factor loading was 0.40 for that factor [47]. The simple structure, interpretability criteria, and at least three to four items per factor were used to interpret a factor solution. The homogeneity of factor solution(s) was determined by calculating item-total correlations and internal consistency by Cronbach’s alpha. As this was an initial phase of questionnaire development and validation using EFA, an alpha of ≥0.6 was regarded as sufficient [48].

### 2.4. Ethics Approval and Consent to Participate

Research and Ethical Committee of the Universiti Sains Malaysia (JEPeM) accorded ethical approval for this research (USM/JEPeM/17010063) on 30 March 2017. The study was carried out conforming to the Declaration of Helsinki. Written consents were obtained from the participants. The respondents were given full freedom in their decision for participation in this study. Their participations in this study were totally voluntary. They were given a liberty to refuse or to stop participation in the study at any time, without a penalty or loss of benefits to which they were otherwise entitled. The study also did not have any affect towards any treatment or services available to them. The data were independent and would not be used for any achievement assessment and decision related to healthcare plan.

### 2.5. Availability of Data and Material

All records from this study were kept confidential. Research records were securely stockpiled in a locked cabinet and study data were saved in a password-protected thumb-drive. The research team was the only party that had access to the study data. The datasets used and/or analyzed during the current study are available from the corresponding author on reasonable request.

## 3. Results

Table 3 demonstrates the demographics of the study participants. Data about smoking, cigarette cessation, their awareness and economics are shown in Table 4.

**Figure 3:**
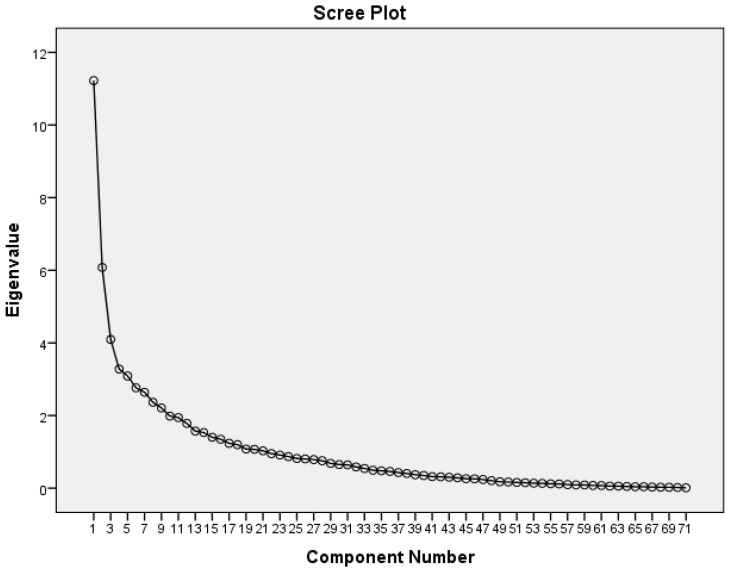
The screen plot for the initial PCA. The slope of the curve vaguely leveled off just after the 7th factor. Using eigenvalue-more-than-1 criteria would definitely take in more than 10 components/factors, which would not blend well the corresponding framework.

An initial principle components analysis (PCA) (a method to identify variables that share similarities) of all the 71 items showed a KMOMSA of 0.501. This is just adequate to indicate sampling adequacy of PCA to produce a reliable estimate. At least two questions were highly correlated as the Bartlett’s test of sphericity was strongly significant (<0.001). Twenty-one factors appeared with an eigenvalue above 1, indicative of acceptable importance, and they cumulatively explain 77.3% of the responses (questionnaire scores) variance. However, when the initial scree plots (another method to assess the suitable number of factors, see Figure 3), it demonstrated that seven factors (which explained 46.7% of the score variance) should be included in the initial model. Section E was taken out of the items pool during initial rotation using direct oblimin method (a method to identify correlated factors) because the result did not optimize (the PCA failed to show important correlations between the factors, see Table 5). Following this, the orthogonal (varimax) rotation method was then used. With varimax rotation, 28 out of 70 items did not load to any factors at all, suggesting a lack of relationship between the factors and the positive smoker identity construct.

Given that multi-dimensional scales should be quintessentially trimmed down to about the same number of items per factor and that too lengthy scales could cause survey fatigue [49], Factor 1 and Factor 2 were further reduced to eight items per factor. The decision was based on the corrected item total correlation results (Table 6 and Table 7).

**Figure 4:**
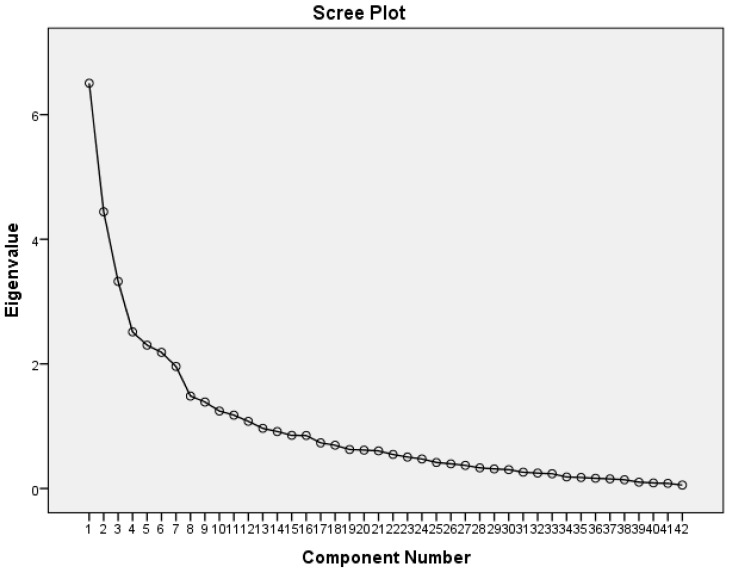
The screen plot for the repeated PCA. A clear demonstration of the elbow of the screen plot. Twelve factors emerged with eigenvalue more than 1. Seven factor-solution would be the best factor solution according the scree plot.

Items with the lowest correlation within Factor 1 (Item A1a, A1f, A1i, A3, A4, A5, A7, A10, A11, A12, and A13) and Factor 2 (Item B9, B14 and B18) were eliminated. PCA was repeated on the remaining 42 items. The result of the repeated PCA showed an improved KMOMSA of 0.608 and a significant Bartlett’s test of sphericity. Twelve factors emerged with an eigenvalue above 1, cumulatively explaining 70.5% of the response variance. The new scree plot (Figure 4) showed that seven factors [which explained 55.3% of the variance] were more acceptable to be included because the slope of the curve was clearly levelling off at that point. The direct oblimin rotation method used in the repeated PCA depicted that there were only very weak correlations at most between the new factors (Table 8). Therefore, the orthogonal (varimax) rotation method was again used. The result is shown in Table 9. This internal factor framework can be regarded as elementary proof for the validity of this instrument.

We calculated coefficient of reliability (Table 10) based on Cronbach’s alpha for each of the seven factors, where values above 0.6 indicate acceptable reliability. Factor 1, 2, 3 and 7 show high reliability but Factor 4, 5 and 6 scored do not (alpha of less than 0.6). 

Next, we show the results of correlations between each of the four factors with Cronbach’s alpha ≥0.6 to *i*) one another (to measure discriminant validity), *ii*) total PSmoQi score (to measure convergent validity), *iii*) preparedness in smoking cessation (Section E), *iv*) FTND score; *v*) CSEQ score (to measure predictive validity), *vi*) SSCS score (to measure concurrent validity), and vii) number of quit attempts. The results are shown in Table 11.

Each of the four factors were discriminated from each other by having low inter-correlation among factors, showing there is significant evidence of divergent validity of the factors. Significant correlations between each of the four factors with the total PSmoQi score provides evidence that all items from the four factors converged on the same positive smoker identity construct, depicting its strong convergent validity. Factor 1 (contributory factors) and Factor 7 (behaviour in relation to smoking) manifested themselves as positive domains whilst Factor 2 (contextual and temporal patterning) and Factor 3 (identity related to smoking) came up as negative domains (reversed-score domain) to PSmoQi construct. Concurrent validity towards preparedness in stopping smoking was significantly demonstrated in Factor 3, although the other three factors in PSmoQi showed low correlations. Factor 3 also showed a strong negative relationship with FTND, indicating its high concurrent validity in anticipating nicotine dependence. Strong negative correlations with CSEQ exhibited by Factor 1 proved this domain’s concurrent validity in determining smokers’ self-efficacy to avoid smoking in various situations. Against a validated and well-established SSCS, the total PSmoQi score (especially for Factor 1) demonstrated a strong concurrent validity, which verified their conceptual relatedness and construct validity of a well-established scale. Nevertheless, only Section E of the PSmoQi questionnaire was strongly related to the number of smoking cessation quit attempts by the smoker within the past year, showing that this “preparedness in stop smoking” item has a strong criterion validity in predicting the efforts of smokers to cease smoking.

## 4. Discussion

Improvement of the total variance explained from 46.7% to 55.3% after a repeated test was crucial. This step was important because Streiner [50] has recommended that factors should explain at least 50% of the total variance. Also, Netemeyer et al. [51] suggested that for any one factor to be considered relevant, at least 5% of the total variance explained should be attributable to that factor. The repeated test also improved the KMOMSA and the scree plot substantially.

We developed and validated the positive smoker identity questionnaire (PSmoQi) for smoking cessation based on a comprehensive review of the literature, existing scales and expert opinions. We measured it against FTND, CSEQ and SSCS, and using validation and a reliability analysis we established that the PSmoQi contains adequate validity (face, content, discriminant, predictive and concurrent validity) and reliability (Cronbach’s alpha). 

The six-item Factor 3 was one of the most powerful domains in the PSmoQi construct because it statistically correlated and possibly predicted two of the most important components in the smoking cessation pathway, namely, preparedness of the smokers to smoking cessation (significant positive correlation), as well as correlating the degree of nicotine dependence at the same time (significant negative correlation). The item “I smoke but I am not a smoker” in the Factor 3 domain showed how a low “identity related to smoking” score was related to high preparedness, willingness and strong desire to quit smoking. A negative correlation between Factor 3 and FTND illustrated that those smokers with a low “identity related to smoking” score was significantly correlated with a lower degree of nicotine dependence. This finding is quite important in smoking cessation because higher a level of nicotine dependence was associated with relapse during cessation attempts [52], and lower levels of dependence consistently correlated with making quit attempts and the success of those attempts [53].

Smoker’s self-efficacy to avoid smoking in various situations played a crucial role in smoking cessation pathway [54,55]. Our results showed that Factor 1 can provide a valid measure in estimating the degree of self-efficacy (CSEQ) among smokers in staying away from smoking. For instance, the items A1g “Smoking causes smokers look more stylish” and A1c “Smoking causes smokers look more popular”, which loaded highest within Factor 1, showed that those who feel positive about smoking may not be able to refrain from it as they will miss the perceived stylishness and the popularity cigarette smoking would bring. 

The smoker self-concept scale measures the self-concept construct in social cognitive conception with an alpha-coefficient of 0.74 [35]. From our extensive literature review, the self-concept construct is the most conceptually similar construct in comparison with the positive smoker identity construct from PRIME theory. Significant concurrent validity, which was demonstrated in our result, verified this resemblance, although the origin of both constructs was distinguishable. 

Sector E provided the only, but most essential, single-item domain in the PSmoQi questionnaire due to its strong criterion validity with CSEQ, SSCS and the number of smoking cessation attempts. A high Sector E score was significantly related to a high CSEQ score. This finding reflected that those who have high efficacy to ward off smoking in various situation would be more prepared and willing to quit compared with those who have lower efficacy. A strong negative correlation between Sector E and SSCS also echoed the concurrent validity of the self-concept construct towards preparedness to quit smoking. According to our finding, those who had a high smoker self-concept score were related to lower preparedness and willingness to quit smoking. This result was parallel to the other studies about self-concept construct and smoking cessation [54,55]. Nevertheless, the most important step in the pathway of smoking cessation was when the preparedness and willingness to quit smoking were translated to the action of attempting to quit itself. The validity of such a relationship was statistically demonstrated by the significant positive correlation of Section E with the number of quit attempts measure. Section E would provide a powerful domain cum instrument for any smoking cessation screening program, either in the clinic or in the community settings. 

However, there is a caveat to this finding. Section E was removed from the items pool after the initial factor extraction process because the item loaded poorly to the overall positive smoker identity construct. This was evident during the direct oblimin rotation phase. The web of complexity of the smoking cessation pathway was broken down further by this finding because we showed that the overall positive smoker identity was not entirely related to preparedness or readiness to quit smoking except a part of it, which was Factor 3 (identity related to smoking). We could safely suggest that Section E, which significantly demonstrated its construct validity in measuring preparedness to stop smoking, could stand as a construct of its own. It is interesting to see that Section E was significantly correlated to SSCS but not to the overall PSmoQi construct. This finding reflects that PSmoQi may possibly be a richer, deeper and broader construct compared to SSCS. The richness and depth of PSmoQi in comparison to SSCS was further evident from SSCS’s significant correlation with Sector E, FTND and CSEQ. Whilst none of these constructs were correlated with the overall PSmoQi construct, these constructs did correlate with one of its four domains, as explained above. 

A further limitation of this study is that all the samples in this study were male. This would actually reflect the smoking prevalence in Malaysia, where the male prevalence of smoking was far greater (43.0%) than female prevalence (1.4%) [56]. Although we tried to conveniently select any smoker irrespective of gender during data collection, male smokers were more willing to participate and consent for the study. Males were more likely to have a positive smoker identity compared to females, according to Tomor et al [36]. This indicates a possible effect of a volunteer bias (selection bias) in this study. In addition, female smokers were probably more negatively evaluated in terms of health, purity, respect, self-control, and good judgment in Kelantan compared to Kuala Lumpur or other cities in the west coast of Peninsular Malaysia, where there are less conservative populations. A larger sample size would probably further improve the factor analysis, as the KMOMSA just narrowly passed 0.5 mark. 

## 5. Conclusions

The initial observation discussed here lends evidence for internal validity and reliability of the PSmoQi questionnaire. The results indicate that the PSmoQi accurately and consistently measures the diverse elements of identity which smokers expressed positively towards cigarette smoking. The advantage of the positive smoker identity construct delivered via the PSmoQi questionnaire was that it contained validated domains which correlated with many components in the smoking cessation pathway. The components comprised the preparedness to stop smoking, nicotine dependence, smoker’s self-efficacy to avoid smoking in various situations, and the smoker’s effort in smoking cessation demonstrated by the number of attempts to quit. The strong relationship between the different domains of positive smoker identity and various other constructs in the smoking cessation pathway conveyed that this construct could possibly be a practically pertinent facet and provide better understanding in helping smokers to quit. The discovery of Sector E as an entirely distinct construct that measures preparedness towards quitting smoking is an enormous bonus. It would provide a useful instrument in identifying those smokers who are actually ready to quit smoking.

Although it is encouraging, more efforts are required in order to delve deeper into the varied fundamental aspects of positive smoker identity, as well as its connection to smoking cessation success. Our research team plans to carry out a future study with a larger sample size to explore more of the positive identity construct using confirmatory factor analysis and regression analysis. It is conceivable that Factor 3 and Factor 4 would load better then, thus improving its validity and overall reliability. Further studies on younger sample of smokers could also provide better understanding on the result of previous study [27], that is, the reason why those denying their smoker identity have not attempted to quit in the past year, and of the young and social smoker paradox [55,57].

Copyright: The authors currently hold a copyright relating to the content of the questionnaire (CR-1 NOTIFICATION OF WORKS: LY2018003267). The authors declare that they do not have any other financial competing interests.

## Figures and Tables

**Table 1:** Smoker identity construct measurements in literatures.

Studies	Population	Smoker Identity Construct	Question(s) Used
Berg et al., (2009) Minnesota, USA [27]	College students	Yes or No	Do you consider yourself a smoker?
Choi et al., (2010) Michigan, USA [28]	University students	Yes or No	Do you consider yourself a smoker?
Levinson et al., (2007) Denver, USA [29]	College students	Yes or No	Do you consider yourself a smoker?
Ridner et al., (2010) Kentucky, USA [30]	College students	Single item response choices.	Which of the following best describes you? (non-smoker, smoker, occasional smoker, and social smoker.
Hertel and Mermelstein, (2012) Chicago, USA [31]	High school students	Two continuous Likert-scale items and a categorical scale item.	1. How much is being a smoker part of who you are? (1 = not at all, to 4 = a lot). 2. How important are cigarettes in your life? (1 = not at all important, to 5 = the most important) 3. Which of the following best describes how you think about yourself? (1 = smoker, 2 = social smoker, occasional smoker, 3 = ex-smoker, 4 = someone who tried smoking, 5 = non-smoker).
Falomir and Invernizzi, (1999) Spain [32]	Secondary school students	Three response scale items. (1 = a little, to 7 = a lot)	1. To what extent do you feel you are a real smoker? 2. To what extent do your friends see you as a real smoker?
Shadel and Mermelstein, (1996) Chicago, USA [35]	Clinic-based smoking cessation programme adult clients	Five-item Smoker Self-Concept Scale (1 = strongly disagree, to 7 = strongly agree)	1. Smoking is part of my self-image. 2. Smoking is part of "who I am." 3. Smoking is a part of my personality. 4. Smoking is a large part of my daily life. 5. Others view smoking as part of my personality
Tombor et al., (2013) UK [36]	National adult survey	Yes or No	I like being a smoker
Tombor et al., (2015) UK [37]	Adult household survey	Yes or No	I still think of myself as a smoker
Meijer et al., (2018) Netherlands [38]	Longitudinal survey	2 items (1 = strongly agree to 5 = strongly disagree)	Two items for smokers and ex-smokers: “to [continue smoking/start smoking again] would fit with who you are” and “to [continue smoking/start smoking again] would fit with how you want to live”

**Table 2:** Four eliminated items from the face and content validity phase.

No.	Items
1.	*Merokok membuatkan seseorang itu kelihatan lebih sasa* (Smoking makes a person looking more sturdy)
2.	*Merokok membuatkan seseorang itu kelihatan lebih seksi* (Smoking makes a person look sexier)
3.	*Merokok membuatkan seseorang itu kelihatan lebih menggoda* (Smoking makes a person look more sexually attractive)
4.	*Merokok dapat membantu saya membuat apa sahaja yang saya mahu* (Smoking can help me do whatever I want)

**Table 3:** Socio-demographic characteristics of the participants.

Variable	N (%)
Mean Age (SD)	38 (9.21)
Sex	
Men	100 (100)
Women	0 (0)
Ethnicity	
Malay	100 (100)
Others	0 (0)
Education level	
Primary school or lower	1 (1)
Secondary school	53 (53)
Certificate or Diploma Level	43 (43)
Bachelor Degree	2 (2)
Master or higher	1 (1)
Marriage Status	
Single	10 (10)
Married	89 (89)
Widower	1 (1)
Income (Ringgit Malaysia;RM) median, (interquartile range)	RM2000 (1500)

**Table 4:** Data about smoking, cigarette cessation, and their awareness.

Variable	N (%)
Smoker type	
Daily	77 (77)
Occasional	23 (23)
Tobacco products consumed	
Conventional cigarette	95 (95)
Vape	12 (12)
Shisha	4 (4)
Pipe	1 (1)
E-cig	1 (1)
Others	1 (1)
Mean age start smoking (SD)	18 (3.78)
Mean age start smoking regularly	21 (3.53)
Frequency of smoking	
Daily	92 (92)
Once a week	6 (6)
Once a month	1 (1)
Less frequent than once a month	1 (1)
No. of cigarette per day	
1 or less	5 (5)
2 to 5	26 (26)
6 to 10	31 (31)
11 to 20	25 (25)
More than 20	13 (13)
Place of smoking	
Home	69 (69)
Workplace	22 (22)
Friend’s house	29 (29)
Food café	61 (61)
Public place	20 (20)
Social gathering	21 (21)
Others	11 (11)
Mean Number of cessation trial in the last 1 year (SD)	1.6 (2.07)
Median number of days stop in the last trial (interquartile range)	3 (7)
Methods of smoking cessation trial	
Never stop	30 (30)
Willpower	54 (54)
Over-the-counter medications	5 (5)
Advice of friends	5 (5)
Health counselling	6 (6)
Professional NRT	2 (2)
Others	4 (4)
Exposure to smoking cessation campaign	
Always	45 (45)
Occasional	51 (51)
Never	4 (4)
Median cost of smoking in Ringgit Malaysia (RM) per month (interquartile range)	RM120 (130)
Usage of cheaper than market price cigarette	
All of them (100%)	36 (36)
Most of them (70% to 99%)	20 (20)
Occasionally (30% to 69%)	21 (21)
Rarely (1% to 29%)	8 (8)
Never	15 (15)

**Table 5:** Correlation matrix table between the new factors during initial PCA.

Component	Factor 1	Factor 2	Factor 3	Factor 4	Factor 5	Factor 6	Factor 7
Factor 1	1.00	−0.06	0.14	0.16	0.07	−0.14	−0.02
Factor 2	−0.06	1.00	0.09	−0.01	0.10	0.15	0.06
Factor 3	0.14	0.09	1.00	0.04	0.06	−0.09	0.01
Factor 4	0.16	−0.01	0.04	1.00	−0.02	−0.09	−0.09
Factor 5	0.07	0.10	0.06	−0.02	1.00	0.04	0.00
Factor 6	−0.14	0.15	−0.09	−0.09	0.04	1.00	0.03
Factor 7	−0.02	0.06	0.01	−0.09	0.00	0.03	1.00

**Table 6:** Factor 1 corrected item total correlation and Cronbach’s alpha if deleted.

Item-Total Statistics					
Item	Scale Mean If Item Deleted	Scale Variance If Item Deleted	Corrected Item-Total Correlation	Squared Multiple Correlation	Cronbach’s Alpha If Item Deleted
A1a	46.76	166.992	0.544	0.488	0.928
A1b	46.34	162.732	0.660	0.677	0.926
A1c	46.66	162.833	0.722	0.685	0.925
A1d	46.31	161.105	0.716	0.639	0.925
A1f	45.71	162.895	0.586	0.583	0.928
A1g	46.45	162.088	0.744	0.684	0.925
A1i	45.98	161.495	0.617	0.657	0.927
A1j	46.08	158.781	0.736	0.719	0.924
A2	46.08	162.377	0.721	0.667	0.925
A3	46.01	163.485	0.622	0.589	0.927
A4	46.15	165.199	0.598	0.520	0.927
A5	46.19	163.489	0.642	0.568	0.926
A6	46.29	161.925	0.684	0.575	0.926
A7	46.33	164.749	0.572	0.512	0.928
A10	45.32	169.371	0.459	0.390	0.930
A11	46.38	165.733	0.564	0.486	0.928
A12	46.47	168.231	0.501	0.624	0.929
A13	46.49	169.222	0.418	0.615	0.931
A14	46.58	163.438	0.685	0.636	0.926

**Table 7:** Factor 2 corrected item total correlation and Cronbach’s alpha if item deleted.

Item-Total Statistics					
Item	Scale Mean If Item Deleted	Scale Variance If Item Deleted	Corrected Item-Total Correlation	Squared Multiple Correlation	Cronbach’s Alpha If Item Deleted
A8	38.79	51.602	0.602	0.688	0.806
A9	38.72	51.072	0.587	0.720	0.806
B3	38.38	51.309	0.538	0.464	0.811
B5	38.93	50.894	0.519	0.304	0.813
B6	38.52	52.111	0.577	0.373	0.808
B7	39.07	54.187	0.467	0.357	0.817
B9	39.02	53.535	0.454	0.583	0.819
B10	38.93	53.116	0.499	0.610	0.815
B12	38.64	53.041	0.508	0.414	0.814
B14	38.86	54.970	0.395	0.335	0.823
B18	39.04	54.685	0.360	0.300	0.827

**Table 8:** Correlation matrix table between the new factors (repeated PCA).

Component	Factor 1	Factor 2	Factor 3	Factor 4	Factor 5	Factor 6	Factor 7
Factor 1	1.000	−0.087	0.072	0.056	0.032	0.037	−0.188
Factor 2	−0.087	1.000	0.089	0.017	−0.011	0.168	0.132
Factor 3	0.072	0.089	1.000	−0.041	−0.027	0.090	−0.122
Factor 4	0.056	0.017	−0.041	1.000	0.000	0.002	−0.049
Factor 5	0.032	−0.011	−0.027	0.000	1.000	−0.018	−0.031
Factor 6	0.037	0.168	0.090	0.002	−0.018	1.000	−0.017
Factor 7	−0.188	0.132	−0.122	−0.049	−0.031	−0.017	1.000

**Table 9:** Exploratory factor analysis (repeated PCA).

Rotated Component Matrix ^a^								Item-Total Correlation ^b^
Item	Factors and Loading							
	Contributory Factors	Contextual and Temporal Patterning	Identity Related to Smoking	Factor 4	Factor 5	Factor 6	Behaviour in Relation to Smoking	
A1b	0.737							0.689
A1c	0.792							0.760
A1d	0.775							0.690
A1g	0.823							0.753
A1j	0.789							0.701
A2	0.707							0.652
A6	0.721							0.655
A14	0.697							0.658
A8		0.797						0.660
A9		0.790						0.661
B3		0.655						0.528
B5		0.645						0.509
B6		0.664						0.563
B7		0.484						0.414
B10		0.458						0.402
B12		0.610						0.476
B8								-
B17				0.632				0.246
B22				0.741				0.155
B23				0.692				0.343
C10				−0.507				−0.275
D13				−0.534	0.444			−0.228
B1								-
B4			0.610					0.453
B13			0.505					0.356
C1			0.648					0.541
C6			0.411					0.408
D7								-
D8			0.653					0.417
D9			0.705					0.541
C5					-0.571			−0.430
D3					0.697			−0.005
D12					0.699			0.053
C2							0.522	0.372
C3							0.688	0.508
C4							0.713	0.485
C13							0.602	0.292
B11						−0.580		−0.042
B20						−0.477		0.053
D5						0.535		0.241
D10						0.692		0.006
D11						0.547		0.280

Extraction method: Principal component analysis; Rotation method: Varimax with Kaiser normalization; a = rotation converged in 14 iterations; b = corrected item-total correlation; computed using only items within factor.

**Table 10:** Coefficient of reliability of the factors (n = 100).

Factors	Coefficient of Reliability (Cronbach’s Alpha)
Contributory factors	0.90
Contextual and temporal patterning	0.81
Identity related to smoking	0.72
Behaviour in relation to smoking	0.65
Factor 4	0.05
Factor 5	−0.45
Factor 6	0.22

**Table 11:** Pearson correlation matrix for the scales.

Scales	Total PSmoQi Scores	Factor 1	Factor 2	Factor 3	Factor 7	Sector E	FTND	CSEQ	SSCS	No. of Quit Attempt
Total PSmoQi Scores	1									
Factor 1	0.683 **	1								
Factor 2	−0.642 **	−0.055	1							
Factor 3	−0.393 **	0.037	0.114	1						
Factor 7	0.268 **	0.127	0.149	−0.193	1					
Sector E	−0.119	−0.132	−0.106	0.238 *	−0.127	1				
FTND	0.169	0.077	−0.051	−0.239 *	0.078	−0.160	1			
CSEQ	−0.188	−0.276 **	−0.001	0.076	0.044	0.248 *	−0.327 **	1		
SSCS	0.516 **	0.545 **	−0.257 **	−0.009	0.057	−0.258 **	0.242 *	−0.375 **	1	
No. of quit attempt	−0.040	−0.070	−0.078	0.131	−0.013	0.235 *	−0.085	0.147	−0.158	1

* *p* < 0.05 (2-tailed); ** *p* < 0.01 (2-tailed); Pearson correlation coefficients (n = 100).
